# The impact of school water, sanitation, and hygiene improvements on infectious disease using serum antibody detection

**DOI:** 10.1371/journal.pntd.0006418

**Published:** 2018-04-16

**Authors:** Anna N. Chard, Victoria Trinies, Delynn M. Moss, Howard H. Chang, Seydou Doumbia, Patrick J. Lammie, Matthew C. Freeman

**Affiliations:** 1 Department of Environmental Health, Rollins School of Public Health, Emory University, Atlanta, Georgia, United States of America; 2 Division of Foodborne, Waterborne, and Environmental Diseases, National Center for Emerging and Zoonotic Infectious Diseases, Centers for Disease Control and Prevention, Atlanta, Georgia, United States of America; 3 Department of Biostatistics, Rollins School of Public Health, Emory University, Atlanta, Georgia, United States of America; 4 Malaria Research and Training Center, Faculty of Medicine and Odontostomatology, University of Sciences, Techniques and Technologies of Bamako (USTTB), Bamako, Mali; 5 Division of Parasitic Diseases and Malaria, Centers for Global Health, Centers for Disease Control and Prevention, Atlanta, Georgia, United States of America; Swiss Tropical and Public Health Institute, SWITZERLAND

## Abstract

**Background:**

Evidence from recent studies assessing the impact of school water, sanitation and hygiene (WASH) interventions on child health has been mixed. Self-reports of disease are subject to bias, and few WASH impact evaluations employ objective health measures to assess reductions in disease and exposure to pathogens. We utilized antibody responses from dried blood spots (DBS) to measure the impact of a school WASH intervention on infectious disease among pupils in Mali.

**Methodology/Principal findings:**

We randomly selected 21 beneficiary primary schools and their 21 matched comparison schools participating in a matched-control trial of a comprehensive school-based WASH intervention in Mali. DBS were collected from 20 randomly selected pupils in each school (n = 807). We analyzed eluted IgG from the DBS using a Luminex multiplex bead assay to 28 antigens from 17 different pathogens. Factor analysis identified three distinct latent variables representing vector-transmitted disease (driven primarily by dengue), food/water-transmitted enteric disease (driven primarily by *Escherichia coli* and *Vibrio cholerae*), and person-to-person transmitted enteric disease (driven primarily by norovirus). Data were analyzed using a linear latent variable model. Antibody evidence of food/water-transmitted enteric disease (change in latent variable mean (β) = -0.24; 95% CI: -0.53, -0.13) and person-to-person transmitted enteric disease (β = -0.17; 95% CI: -0.42, -0.04) was lower among pupils attending beneficiary schools. There was no difference in antibody evidence of vector-transmitted disease (β = 0.11; 95% CI: -0.05, 0.33).

**Conclusions/Significance:**

Evidence of enteric disease was lower among pupils attending schools benefitting from school WASH improvements than students attending comparison schools. These findings support results from the parent study, which also found reduced incidence of self-reported diarrhea among pupils of beneficiary schools. DBS collection was feasible in this resource-poor field setting and provided objective evidence of disease at a low cost per antigen analyzed, making it an effective measurement tool for the WASH field.

**Trial registration:**

The trial was registered at ClinicalTrials.gov (NCT01787058)

## Author summary

Water, sanitation, and hygiene (WASH) in schools is promoted as an intervention to improve child health in low-resource settings. However, evidence of the impact of school WASH interventions on child health is mixed. One reason could be that most studies rely on self-reported disease symptoms, which are prone to bias. In order to objectively measure evidence of disease, we collected dried blood spots (DBS) from pupils attending schools participating in an impact evaluation of a comprehensive school WASH intervention in Mali, and analyzed the DBS for antibody responses to 28 antigens from 17 different pathogens. We found that evidence of enteric disease was lower among pupils attending beneficiary schools compared to pupils attending comparison schools. These results are consistent with those from the parent study, which also found reduced self-reported diarrhea among pupils attending beneficiary schools. Our results support WASH in schools as an effective intervention to improve child health. Further, DBS are a feasible measurement tool for the WASH field to provide objective evidence of disease.

## Introduction

Diarrhea is among the leading causes of morbidity and mortality among children in developing communities [1], and children are often disproportionately affected by other infectious diseases, including soil-transmitted helminths (STHs) and trachoma [2]. The role of improvements in household safe water, sanitation access, and hygiene (WASH) behaviors on the reduction of infectious diseases among children and adolescents is well documented [1, 3–5]. Despite the biological plausibility supporting the role of improvements in school WASH conditions on pupil health, results from school WASH evaluations have been mixed [6–12]. There is some evidence of associations between WASH in schools programs and reductions in diarrhea, acute respiratory infection, soil-transmitted helminth re-infection, and school absence, but results are inconsistent and effects are sometimes evident only among sub-populations [6, 8, 9, 13–17].

One limitation to health impact evaluations of WASH interventions in low-resource settings is the existing methods and tools used to measure diarrheal and other infectious disease incidence. A common approach for measuring diarrheal disease is self-report, an approach prone to recall and social desirability biases. Respondents’ variable and often subjective interpretations of the definition of “diarrhea” may also lead to imprecise measurements of incidence [18, 19]. Further, the definition of “diarrhea” and the recall period are not uniform across studies [18, 20, 21], making inter-study comparison of disease incidence and intervention effectiveness difficult. Stool collection is another, more accurate approach to assessing enteric infections, however, conventional methods lack sensitivity for many pathogens [22]. The cost and logistical implications for this approach are considerable. Stool must be collected, often by return visit, and samples must be transported to laboratory equipment and trained laboratory personnel for the identification of pathogenic agents [22, 23], making it a challenging and expensive way to assess infectious disease prevalence in low-resource field settings.

Antibody detection assays are used to detect immune responses to past infections from a variety of different organisms by detecting signal intensity due to the presence of antibodies [24–28]. Luminex multiplex bead assay (MBA) technology detects antibodies in a range of biological specimens including eluted antibodies from dried blood spots (DBS) obtained through a single finger-prick. As such, this technology has significant potential for providing reliable measures of infections in low-resource settings, as DBS samples are stable at ambient temperatures and can be collected, transported, and stored easily, negating the need for expensive equipment and skilled laboratory technicians in the field [23, 29]. Because the multiplexing capacity allows for the simultaneous analysis of up to 100 different antigens from one sample, and because samples can be analyzed off-site in a reference laboratory, Luminex MBAs have been shown to be an effective method for data collection in low-resource settings and at a low cost per antigen analyzed. Previous studies have used Luminex MBAs to detect serum antibody responses to tuberculosis, lymphatic filariasis, chikungunya, dengue, malaria, and enteric protozoa (*Giardia intestinalis*, *Entamoeba histolytica*, and *Cryptosporidium parvum*) [25–27, 30–32].

We collected DBS to evaluate the impact of a school WASH program in Mali on infectious disease by analyzing immunoglobulin (Ig) G responses to 28 antigens from 17 different pathogens using the Luminex MBA platform. Although this serological platform has been widely used to evaluate drug treatment programs [25, 31] and as a disease diagnostic and surveillance tool [26, 27, 33], it has had limited employment within WASH program impact evaluations [34]. In addition to providing evidence for the impact of school WASH interventions on pupil health, data from this study also provide evidence for the feasibility of using the Luminex platform as an objective measurement of enteric pathogen exposure. Further, this study highlights the benefit of utilizing the Luminex MBA platform’s multiplexing capacity to simultaneously assess serological episodes for a range of infectious diseases.

## Methods

### Ethics

This study was approved by the Ethics Committee of the National Institute of Public Health Research in Mali (*Comité d’Ethique de l’Institut National de Recherché en Santé Publique*, 02/2014/CE-INRSP) and the Institutional Review Board of Emory University (IRB00060756). The trial was registered at ClinicalTrials.gov (NCT01787058). We obtained informed written consent (signature or fingerprint) from the parents of all participants and oral assent from all participants prior to any interview data or blood spot collection. Laboratory staff from the United States Centers for Disease Control and Prevention had no contact with children nor access to personal identifiers.

### Setting

This study was nested within a longitudinal impact evaluation of the Dubai Cares Water, Sanitation, and Hygiene in Schools Initiative in Mali (DCIM WASH) project, a comprehensive school-based WASH intervention in 900 schools in Bamako Capital District and the Koulikoro, Mopti, and Sikasso regions of Mali. Using stratified random sampling based on region, a subset of 21 of 100 beneficiary primary schools from the impact evaluation were selected for inclusion, as well as their 21 matched comparison schools, for a total of 42 schools participating in the study (**S1 Fig**). Matched comparison schools were located within the same educational district and matched to beneficiary schools based on baseline enrollment size and school WASH characteristics. Detailed methods of the parent study and the as-treated analysis are described elsewhere [14, 17]. In each school, 20 pupils were randomly selected from a list of all pupils enrolled in classes 1–6 using stratified random sampling based on pupil sex and grade. Pupils were interviewed about their household WASH access, school absence, and recent illness. Capillary whole blood in the form of a dried blood spot (DBS) was collected from each pupil. Data from a total of 807 participants aged 4–17 years were collected between January and May 2014.

### Dried blood spot collection

Students’ ring or middle fingers were cleaned and sanitized with an alcohol wipe, allowed to air dry, and then punctured with a new single-use lancet. The first drop of blood was wiped away [35, 36]. Fingertip whole capillary blood specimens were then collected onto a filter paper wheel with six circular extensions (TropBio Pty Ltd, Townsville, Queensland, Australia), each designed to absorb 10 μl of whole blood. One filter paper wheel was collected per child. The filter paper wheels were air dried for up to 4 hours and placed in a sealed plastic bag with a desiccant. Between 1–3 months following collection, samples were shipped to a laboratory at the Centers for Disease Control and Prevention in Atlanta, Georgia for storage at -20 °C and analysis [35, 36].

### Antigen coupling and antibody analysis

Purified antigens were coupled in various buffers as indicated in **S1 Table**, and some antigens were linked with glutathione-S-transferase (GST). For each antigen and GST, carboxyl groups on the surface of specifically classified-spectral magnetic polystyrene microspheres (MagPlex Beads; Luminex Corporation, Austin, TX) were converted to reactive esters using the 1-ethyl-3-(3-imethylaminopropyl) carbodiimide method (Calbiochem, Woburn, MA). The esters readily react with available primary amine structures on the antigens to form a covalent amide bond between antigen and bead. Coupling efficiency was determined using sera and reagent known to be highly reactive to the antigens and GST.

One circular extension from each child’s DBS wheel was placed in 0.5 mL of elution buffer consisting of PBS with 0.5% bovine serum albumin, 0.3% Tween 20, 0.1% sodium azide, 0.5% polyvinyl alcohol, 0.8% polyvinylpyrrolidone, and 0.1% casein, and allowed to elute overnight at 4 °C. Afterward, the elution was further diluted 1:4 with the same elution buffer, containing sufficient amounts of crude *Escherichia coli* extract for a final concentration of 3 μg/mL. The *E*. *coli* extract is used to absorb *E*. *coli* antibodies that could react with any extraneous *E*. *coli* proteins coupled to the beads. After overnight storage at 4 °C, the eluate was exposed to antigen-coupled beads for 1.5 hours at room temperature. Bound antigen-specific IgG was detected on the coupled beads as previously described [26]. Between steps, the magnetic beads were washed three times with 0.05% Tween 20 PBS, using a Bio-Plex Pro II Wash Station (Bio-Rad, Hercules, CA). Data were acquired using a Bio-Plex 100 reader with Bio-Plex Manager 6.1 software (Bio-Rad). For each antigen, the median fluorescence intensity (MFI) with a range of 1–32,766 channels were determined, and the average from duplicate wells was obtained. From a primary antibody blank, background (bg) was subtracted (MFI-bg) and used as data.

### Measures and statistical analysis

#### Univariate analysis

To measure pupils’ household WASH access, we created an index score using pupil responses to three survey questions on their household access to 1) an improved drinking water source, classified according to the Joint Monitoring Programme definition [37]; 2) any on-site sanitation facility; and 3) soap for handwashing. Affirmative responses were assigned one point and all responses were summed, creating an index score ranging from 0 (no household WASH access) to 3 (maximum household WASH access).

Differences in pupil demographics (age, sex, grade) and household WASH access by intervention status were evaluated using logistic (sex) and linear regression models (age, grade, household WASH access), with random intercepts at the school level. Associations with a *p* <0.05 were considered statistically significant.

#### Factor analysis and latent variable development

Factor analysis is a statistical tool commonly used in behavioral and health sciences to assess complex inter-relationships among large numbers of variables, including non-independent or correlated variables. The theory behind factor analysis is that multiple observed variables with similar patterns of response are all associated with an underlying latent variable. Thus, the goal of factor analysis is to yield a small number of new variables (factor constructs) that adequately express the communality of–and can substitute for–a larger number of variables [38, 39]. Given that we had data on antibody responses from 28 different antigens for infectious disease, some of which may be correlated, we employed factor analysis to identify latent variables representing groupings of antibody responses.

The 28 variables representing antibody responses to infectious diseases were normalized by taking the natural log and standardized [40]. We used an iterative approach, specified *a priori*, for selecting antibody response variables to include in the factor analysis. First, we restricted the analysis to include only antibody responses that were prevalent in the population; all unique antibody response variables for which a cutoff value for infection was available and for which <10% of samples exceeded the cutoff value were excluded (chikungunya, *Brugia malayia*, *Wuchereria bancrofti*, *Taenia solium*, and yellow fever). The following unique antibody response variables were included in the initial factor analysis: *E*. *histolytica*, *G*. *intestinalis* VSP3, *G*. *intestinalis* VSP5, *Plasmodium falciparum* MSP-1_19_, *P*. *falciparum* MSP-1_42_, *P*. *falciparum* AMA-1, *P*. *vivax* MSP-1_19_, entero-toxigenic *E*. *coli* (ETEC), *V*. *cholerae*, Dengue 2, Dengue 3, norovirus GI.1 (Norwalk strain), norovirus GII.4 (Sydney strain), norovirus GIV.1 (St. Cloud strain), *Cryptosporidium parvum* 17-kDa, *C*. *parvum* 27-kDa, *Schistosoma mansoni*, *Campylobacter jejuni* p18, *C*. *jejuni* p39, *Salmonella typhimurium*, *S*. *enteritidis*, *Chlamydia trachomatis* Pgp3, and *C*. *trachomatis* CT694. Second, we evaluated antigen response variables for uniqueness, a measure of the variance that is not shared with other variables in the model; the higher the uniqueness, the lower the relevance of the variable in the factor model [38, 39]. Since the goal of factor analysis is to identify groupings of variables with similar responses, variables with uniqueness ≥0.6 (thus implying the majority of the variable’s response is not shared with other variables in the model) were dropped from the factor analysis until a reduced factor model consisting only of variables with uniqueness <0.6 was achieved. An oblique rotation was applied given the assumption that factors were correlated, and factors with Eignenvalues >1 were retained [38, 39, 41]. The rotated factor loadings from the reduced factor model became latent variables representing disease responses; from here forward, we refer to these latent variables as disease response variables.

#### Latent variable models

We elected to use a latent variable modeling approach over a more conventional modeling strategy, such as linear regression, because latent variable models allow multiple, correlated outcomes (disease response variables) to be analyzed simultaneously in one model rather than running individual models for each outcome [42, 43]. The association between disease outcomes and intervention status were analyzed using the generalized linear latent and mixed model (gllamm) package [44] in Stata version 13 (StataCorp, College Station, TX).

The latent variable modeling framework consisted of 1) a measurement model of the child-specific latent variables identified through factor analysis, clustered at the school level, and 2) a structural model of the regression of the intervention on the latent variables, controlling for pupil grade, sex, and household WASH access. Pupil grade was included as a proxy for pupil age due to a large number of missing pupil age data (n = 338). Associations with a *p* <0.05 were considered statistically significant.

#### Linear regression models

To cross-validate the linear latent model results, we independently evaluated differences in antibody responses between pupils attending intervention versus comparison schools for each antigen response included in the initial factor model using mixed effects linear regression models with random intercepts at the school level. To facilitate comparison between the linear regression model results and the latent model results, the normalized antibody response variables were used as the outcomes and the same control variables—pupil grade, sex, and household WASH access—were included. Because we *a posteriori* used linear regression to cross-validate the linear latent model results, we included a Bonferroni correction to adjust for multiple comparisons. Associations were considered statistically significant if they had a *p<*0.002, the alpha necessary to reach 95% significance with 23 hypotheses.

## Results

### Bead coupling efficiency

All coupled beads showed high MFI-bg from sera or reagents known to be highly reactive to the antigens, indicating sufficient antigen coupling and the excellent condition of the DBS.

### Student characteristics

DBS were collected from 807 primary school students attending 42 schools (21 beneficiary, 21 comparison). Survey data from 7 pupils, all attending the same beneficiary school, were not collected and these pupils were subsequently dropped from analysis. The final sample population was 800 students. There were no significant differences in age, sex, grade, or household WASH access between beneficiary and comparison groups (**Table 1**). Demographic characteristics of the students were similar to those in the full parent study [14, 17].

**Table 1:** Demographic characteristics of study population.

	Beneficiary (n = 393) Mean (SD) or n(%)	Comparison (n = 407) Mean (SD) or n(%)	*p*^1^
Age^2^	10.9 (0.14)	11.1 (0.17)	0.56
Female	183 (46.6%)	188 (46.2%)	0.93
Grade	3.8 (0.08)	3.9 (0.08)	0.65
Household WASH access scale index score	2.1 (0.03)	2.3 (0.03)	0.28

^1^Differences across strata were evaluated using logistic (sex) and linear (age, grade, household WASH access) regression models, with random intercepts at the school-level. P<0.05 was considered statistically significant.

^2^Age missing for 142 pupils in beneficiary group and 196 pupils in comparison group

### Factor analysis

The final factor model included antibody response variables for ETEC, V. *cholerae*, Dengue 2 VLP, Dengue 3 VLP, norovirus Norwalk strain, and norovirus St. Cloud strain. This factor model resulted in the development of 3 distinct factors, or disease response variables (**Table 2**).

**Table 2:** Rotated factor loadings and unique variances.

Antigen	Factor 1	Factor 2	Factor 3	Uniqueness
ETEC	-0.0112	0.8720	-0.0124	0.2425
*Vibrio cholerae*	0.0046	0.8701	0.0146	0.2402
Dengue 2	0.8760	-0.0223	0.0324	0.2376
Dengue 3	0.8710	0.0161	-0.0341	0.2345
Norovirus Norwalk strain	-0.0481	-0.0493	0.7426	0.4449
Norovirus St. Cloud strain	0.0477	0.0524	0.7422	0.4418

A factor loading can be interpreted as a Pearson correlation coefficient between the original variable and the factor. Factor 1 was strongly correlated with Dengue 2 (0.876) and Dengue 3 (0.871). Based on these variables loading highly with Factor 1, and given that Dengue is transmitted by mosquitoes [45], we classified Factor 1 as a latent variable representing vector-transmitted disease. Factor loadings for ETEC and *V*. *cholerae* were strongly correlated with Factor 2 (ETEC = 0.872, *V*. *cholerae* = 0.871). Given that ETEC and *V*. *cholerae* are transmitted when food or water are contaminated with feces [45, 46], we classified Factor 2 as a latent variable representing food/water-transmitted enteric disease. Lastly, norovirus Norwalk and St. Cloud strains were strongly correlated and loaded most highly with Factor 3 (Norwalk = 0.7426, St. Cloud = 0.7422). Norovirus infection occurs by ingesting stool or vomit from an infected person. Although foodborne and waterborne transmission is possible, norovirus is considered primarily a person-to-person transmitted disease [47–49]; as such, we classified Factor 3 as a person-to-person transmitted enteric disease latent variable.

### Linear latent model results

Results from the linear latent model indicate that there was a 0.24 reduction in the latent variable mean of food/water-transmitted enteric disease, and a 0.17 reduction in the latent variable mean of person-to-person transmitted enteric disease among pupils attending beneficiary schools versus pupils attending comparison schools (**Table 3**). We found no difference in the evidence of vector-transmitted disease between pupils attending beneficiary versus comparison schools (β = 0.11, p = 0.141).

**Table 3:** Linear latent model results of the association between the school WASH intervention and disease response variables.

	β	95% CI	*p*
Vector transmitted disease	0.11	(-0.05, 0.33)	0.141
Food/water transmitted enteric disease	-0.24	(-0.53, -0.13)	<0.001
Person to person transmitted enteric disease	-0.17	(-0.42, -0.04)	0.019

β represents change in latent variable mean

Model controls for pupil age, grade, and household access to WASH and includes a random intercept for school

*p*<0.05 is considered statistically significant

### Linear regression model results

Results from the linear regression models are similar to those from the linear latent model. Among the antibody response variables included in the linear latent model, Dengue 2 and Dengue 3 (antigens making up the vector transmitted disease latent variable) were higher among the intervention group; results for Dengue 3 were not significant once we applied the Bonferroni correction (β = 0.29, p = 0.02). Antigen responses for ETEC and *V*. *cholerae* (food/water transmitted enteric disease) and the two Norovirus strains (person to person transmitted enteric disease) were lower among the intervention group, but not statistically significant. Among antibody response variables not included in the linear latent model, only *C*. *trachomatis* (CT-694) was higher among the intervention group (β = 0.39, p < 0.001) (**Table 4**).

**Table 4:** Linear regression model results of the association between the school WASH intervention and antibody responses.

	β	95% CI	*p*
*Campylobacter jejuni* (P18 Antigen)	0.02	-0.20, 0.24	0.88
*Campylobacter jejuni* (P39 Antigen)	-0.12	-0.31, 0.06	0.18
*Cryptosporidium parvum* (17 KdA Antigen)	0.29	0.09, 0.49	0.01
*Cryptosporidium parvum* (27 KdA Antigen)	0.09	-0.11, 0.28	0.41
Dengue 2	0.09	-0.21, 0.39	0.55
Dengue 3	0.29	0.04, 0.54	0.02
*Entamoeba histolytica*	-0.06	-0.31, 0.20	0.65
*Escherichia coli*	-0.18	-0.40, 0.05	0.12
*Giardia intestinalis* (VSP 3)	-0.02	-0.20, 0.16	0.84
*Giardia intestinalis* (VSP 5)	-0.19	-0.37, -0.01	0.04
Norovirus (Norwalk strain)	-0.01	-0.24, 0.22	0.92
Norovirus (St. Cloud strain)	-0.02	-0.25, 0.22	0.88
Norovirus (Sydney strain)	0.12	-0.28, 0.04	0.14
*Plasmodium falciparum* (MSP_19_)	0.07	-0.13, 0.27	0.51
*Plasmodium falciparum* (MSP_42_)	0.16	-0.13, 0.46	0.29
*Plasmodium falciparum* (AMA_1_)	0.16	-0.20, 0.52	0.38
*Plasmodium vivax* (MSP_19_)	0.14	-0.06, 0.34	0.16
*Salmonella enteritidis*	0.10	-0.05, 0.25	0.20
*Salmonella typhimurium*	0.06	-0.11, 0.23	0.50
*Schistosoma mansoni*	0.22	-0.02, 0.45	0.07
*Chlamydia trachomatis* (CT-694)	0.39	0.20, 0.58	<0.001
*Chlamydia trachomatis* (Pgp3)	0.15	-0.03, 0.33	0.10
*Vibrio cholerae*	-0.07	-0.27, 0.13	0.49

Models control for pupil age, grade, and household access to WASH, and include a random intercept for school

Shaded rows represent antigens included in the final linear latent model

Due to the Bonferroni correction for multiple comparisons, *p*<0.002 is considered statistically significant

## Discussion

This study utilized dried blood spots and the Luminex MBA as a tool to evaluate the impact of a school WASH intervention in Mali on infectious disease among pupils. Antibody evidence of both food/water-transmitted enteric disease and person-to-person transmitted enteric disease was lower among pupils attending beneficiary schools, while the intervention had no impact on antibody evidence of vector-transmitted disease. This study was innovative in its use of antibody responses from DBS to measure the impact of a WASH program. Additionally, utilizing factor analysis on antibody responses to identify latent groupings of disease is a novel approach to the analysis of antibody data.

Consumption of microbiologically safe drinking water, handwashing with water and soap, and use of sanitation facilities that safely contain feces are all strategies for stopping enteric disease transmission along the fecal-oral route. Indeed, we found evidence that food/water-transmitted enteric disease and person-to-person transmitted enteric disease was lower among pupils attending schools benefitting from a comprehensive WASH intervention compared to those attending comparison schools, supporting the idea that school WASH can interrupt disease transmission among pupils. These results also corroborate results from the longitudinal parent study, which found a 29% reduction in the odds of reported symptoms of diarrhea among pupils attending beneficiary schools compared to pupils in the comparison schools [14], and a 35% reduction in the odds of diarrhea among pupils attending beneficiary schools that met all WASH targets compared to pupils attending beneficiary schools that met none of the WASH targets [17]. Additionally, these results contribute to the growing body of evidence supporting the association between WASH in schools and reduced pupil diarrheal incidence [9, 13, 14, 17] and other poor health outcomes [8, 13–17].

We found no impact of the intervention on evidence of vector-transmitted disease, which is more commonly linked to environmental conditions than to WASH access, and is generally controlled through the use of insecticides and elimination of breeding sites [50–52]. Given that a school WASH intervention is unlikely to alter the transmission pathways of vector-borne disease [51], our finding that the intervention did not have a significant impact on evidence of vector-transmitted disease is not surprising. There is little biologic plausibility of an impact of a school WASH program on vector-transmitted disease and indeed we found none; as such, the absence of an impact of the intervention on vector-transmitted disease supports the validity of our findings on enteric infections, and can be considered a negative control.

This study employed novel methodology in the use of antibody data to assess the impact of a WASH intervention. The Luminex MBA serologic platform has had limited use as a WASH program impact evaluation tool. We found that the collection of capillary blood in the form of DBS was feasible in a low-resource field context and acceptable by participants and their guardians and therefore serves as a viable alternative to current methods of biological assessment of WASH-related disease such as stool collection or venipuncture that are labor-, time-, and cost-intensive. Additionally, our results suggest that objectively measuring WASH-related disease might be useful for identifying biomarkers that could serve as proxies for access to WASH. Further, given the multiplexing capacity of the Luminex technology, we were able to capitalize on the DBS antibody data collected for the purpose of the WASH program impact evaluation by including antibody measures for diseases beyond the scope of the program–such as lymphatic filariasis, measles, tetanus, and rubella–at a minimal additional cost; with a total of 36 antigens included in the assay, the cost was ~USD $0.54 per antigen/sample, excluding the costs of labor and antigens. Ongoing sub-analyses from this data are providing valuable information on the effectiveness of mass drug administration [25] and vaccination programs, and could identify areas where these programs have been successful or should be scaled up; additional analyses examine patterns of malaria [27] and neglected tropical disease [25, 53] transmission.

This study also employed novel analysis methods for antibody data. Factor analysis is commonly used in behavioral, health, and life sciences [38, 39]. However, our use of factor analysis on antibody responses to classify latent variables of disease responses is a novel approach. It is important to emphasize that while we labeled each factor as vector-transmitted disease, food/water-transmitted enteric disease, and person-to-person transmitted enteric disease, we did not select how the original antibody response variable loaded into each factor/disease response variable. The finding that the original antibody response variables loaded into three distinct pathways of disease transmission validates our use of factor analysis, which assumes that variables share a common factor due to their similar patterns of response. The validity of this method is also highlighted by the antibody response variables that dropped out of the factor model. For example, pathogens such as *C*. *trachomatis* and *S*. *mansoni* have largely unique transmission vectors and intermediate hosts relative to the other pathogens retained in the model (flies and snails, respectively) [45], contributing to a high unique variance (“uniqueness”) and subsequent elimination from the model. Like dengue, malaria is also a vector-borne disease. However, the high uniqueness of the malarial pathogens (*P*. *falciparum* MSP-1_19_, *P*. *falciparum* MSP-1_42_, *P*. *falciparum* AMA-1, *P*. *vivax* MSP-1_19_) in the factor model could be explained by the extremely high prevalence of malaria in this population; nearly all children had antibody responses exceeding the cutoff values for *P*. *falciparum* (78.4%, 90.7%, 91.6%, respectively), with little variance in antibody response (**S2 Fig, S2 Table**). While there was greater variance in antibody response for *P*. *vivax*, it may have dropped out of the factor model due to different dengue and *P*. *vivax* mosquito vectors (*Aedes aegypti* and *Anopholes*, respectively) and vector behaviors (day biters and night biters, respectively) [45]. Lastly, while *E*. *histolytica*, *Giardia*, *Campylobacter*, *Salmonella* and *Cryptosporidium* share a similar transmission pathway to that of ETEC and *V*. *cholerae* (food/water) [45], these pathogens also dropped out of the factor model. Antibody reactivity for *Campylobacter*, *E*. *histolytica*, *Giardia*, and *Salmonella* are particularly predominant in the first few years of life, and wane thereafter [26, 54, 55], a trend that is also evident in our sample (**S2 Fig**). These variables likely dropped out of the factor model given low antibody responses among our school-aged participants. *Cryptosporidium* antibody response is not associated with age [26], but it is less pathway-specific than other antigens in the model; for example, in addition to the fecal/oral route, *Cryptosporidium* can also be transmitted via inhalation [56, 57], which could explain the high uniqueness of *Cryptosporidium* responses.

Results from the linear models of association between intervention status and antibody responses further strengthen the linear latent modeling approach. In these models the trends for all antibody response outcomes were in the same direction as their respective latent variable. The Dengue 3 outcome was only statistically significant at p<0.05 and prior to the use of the Bonferroni correction. This suggests that analyzing the antigens simultaneously—as is done in the linear latent model—may give us more power to detect an effect as opposed to running each outcome individually. Further, because linear latent models allow multiple outcomes to be analyzed simultaneously, they also eliminate the need for a multiple comparisons correction [42, 43]. Of the antibody response variables included in the linear latent model, Dengue 3 (p = 0.02) was only significant prior to the Bonferroni correction. It is possible that this association was due to a Type I error, especially given that there is little biological plausibility that a school-based WASH intervention would lead to increased incidence of dengue. Indeed, under the Bonferroni correction, the p-value needed to be <0.002 to achieve statistical significance. All but one of the antibody response variables that were eliminated from the factor model were statistically insignificant in the linear model results. Antibody response for *C*. *trachomatis* (CT-694) was significantly higher among pupils attending intervention schools. There is some evidence that WASH in schools interventions have the potential to *increase* exposure to fecal pathogens when the intervention is incompletely delivered or adherence is low. An evaluation in Kenya found that in a trial where sanitation was provided at schools, but handwashing was poor, children had higher fecal hand contamination than children at schools without new sanitation facilities. Researchers hypothesized that pupils’ increased use of toilets led to higher fecal contamination, but that a lack of handwashing behaviors put children at risk [15]. Thus, it is possible that *C*. *trachomatis* (CT-694) was indeed higher among beneficiary schools, considering that fidelity and adherence to the intervention was varied [17]. However, it is more likely that this was a spurious association given that there was no significant difference in *C*. *trachomatis* (Pgp3).

We found that factor analysis was useful in identifying common patterns of disease response in our study population. Future studies examining multiple, and possibly correlated disease outcomes should consider the factor analysis approach as a complement to more conventional modeling techniques. By focusing on the underlying phenomena driving the measured results, factor analysis allows researchers to generalize their findings to a larger measurement domain and improve practical applicability.

### Limitations

There are a number of limitations to this study, mostly associated with the use of antibodies as a measure of infection. First, the assay may detect antibodies for infections that were asymptomatic, which may lead to an over-estimation of morbidity. Second, we measured antibody levels for enteric disease among children over 5 years old, who may have already been repeatedly exposed to a variety of pathogens and developed effective immune responses other than IgG. An example of this may have been shown in a study showing IgG responses to these same *Giardia* antigens that decreased in children > 4.5 years of age [26]. Other immune arms, such as cell-mediated immune responses may allow a more rapid clearing of the antigen and shorter IgG responses. However, these two limitations would likely be similar across beneficiary and comparison groups, thus biasing the estimate towards the null. Third, antibody kinetics vary by pathogen, and the current cross-sectional analysis may have captured antibody responses from infections that occurred prior to the intervention. This could have caused us to underestimate the protective benefit of the WASH program.

There are also limitations associated with the use of factor analysis and linear latent models. Our three measures of disease response are factor variables, and the beta coefficient represents a change in the latent variable mean. As such, the model measures a larger construct than the original antibody response variables, and we are not able to calculate the odds or risk ratios for reductions in specific diseases associated with the intervention. Also, many antibody response variables dropped out of the factor model due to low prevalence or a high unique variance. While there is limited biological plausibility of a WASH intervention impacting transmission of some of these pathogens (e.g. chikungunya, lymphatic filariasis, yellow fever), other pathogens, such as schistosomiasis and trachoma, have been directly linked to WASH access [58, 59]. It is possible that these pathogens could have been impacted by the WASH intervention, but were not included in the final analysis. Another limitation is that not all antigens have a known cut-off value for infection, so whether the original antibody response variable exceeded the cutoff for disease was not taken into consideration when constructing the factors. Lastly, linear latent models assume that the latent variables arise from a normal distribution, which is difficult to verify.

### Conclusions

Our results describe evidence of infectious disease among pupils attending schools benefitting from a comprehensive school WASH program in Mali compared to pupils attending matched comparison schools. We found that evidence of enteric disease (both food/water-transmitted and person-to-person transmitted) was lower among pupils attending beneficiary schools, results which are supported by the parent study, which found reductions in self-reported diarrhea among pupils attending beneficiary schools compared to pupils attending comparison schools. Collecting accurate data on biologic evidence of infectious disease in low-resource field settings can be logistically challenging, expensive, and laborious. We collected DBS and analyzed pupil antibody response for 28 antigens from 17 pathogens using a Luminex MBA, a method that has had limited employment in evaluation of WASH interventions. Our study demonstrates the feasibility and applicability of this method in the WASH field as an objective measure of disease.

## Supporting information

S1 Table Purified antigen, antigen format, amount used in coupling, GST linked, and coupling buffer. (DOCX)

S2 Table Prevalence of enteric and neglected tropical diseases among primary school children in Mali (n = 800). (DOCX)

S1 Fig Map of study school locations. Light gray regions were included in study sample; dark gray regions were not included. Dark gray circles indicate location of beneficiary schools; white circles indicate location of comparison schools.
(TIF)

S2 Fig Mean pupil antibody response by grade level. Red line indicates cut-off value for infection (if it exists).
(TIF)

S1 Dataset (XLSX)
