# Supplementary material for: The impact of school water, sanitation, and hygiene improvements on infectious disease using serum antibody detection
Source: PLoS Negl Trop Dis. 2018 Apr 16;12(4):e0006418. doi: 10.1371/journal.pntd.0006418 (PMC5919668; doi:10.1371/journal.pntd.0006418)
Supplement: S1 Table — (DOCX) [file pntd.0006418.s001.docx]

| **S1 Table.** Purified antigen, antigen format, amount used in coupling, GST linked, and coupling buffer. | | | | | | |
| --- | --- | --- | --- | --- | --- | --- |
| **Pathogen** | **Antigen** | **Format^*^** | **GST Linked** | **Coupling Amount (µg)^¶^** | **Coupling Buffer^£^** | **Ref.** |
| Chikungunya | Envelope-1 Mutant A226V | Recomb. | No | 8.7 | A | [1] |
| *Entamoeba histolytica* | Lectin Adhesion Molecule (LecA) | Recomb. | No | 35.0 | B | [2] |
| *Giardia intestinalis* | Variant Surface Protein 5 | Recomb. | Yes | 35.0 | B | [2] |
| *Giardia intestinalis* | Variant Surface Protein 3 | Recomb. | Yes | 35.0 | B | [2] |
| *Plasmodium falciparum* | Merozoite Surface Protein 1_19_ | Recomb. | Yes | 23.0 | B | [3] |
| *Plasmodium falciparum* | Merozoite Surface Protein 1_42_ | Recomb. | No | 17.0 | B | [4] |
| *Plasmodium falciparum* | Apical Merozoite Antigen 1 | Recomb. | No | 23.0 | B | [4] |
| *Plasmodium vivax* | Merozoite Surface Protein 1_19_ | Recomb. | Yes | 25.0 | B | [4] |
| -------------------- | Glutathione-*S*-transferase | Recomb. | ---- | 15.0 | B | [5] |
| *Brugia malayia* | Bm14 | Recomb. | Yes | 139.0 | A | [5] |
| *Wuchereria bancrofti* | Wb123 | Recomb. | No | 139.0 | A | [5] |
| *Escherichia coli* | Enterotoxigenic β subunit | Recomb. | No | 34.8 | B | [6] |
| *Vibrio cholera* | α and β subunit | Native | No | 34.8 | B | [7] |
| Dengue 2 | Envelope Structure | VLP | No | 52.3 | A | [1] |
| Dengue 3 | Envelope Structure | VLP | No | 34.8 | A | [1] |
| Yellow fever | Envelope Structure | VLP | No | 34.8 | A | [8] |
| Norovirus | Envelope Structure GIV.1 (St. Cloud strain) | VLP | No | 34.0 | A | [9] |
| Norovirus | Envelope Structure GII.4 (Sydney strain) | VLP | No | 36.0 | A | [9] |
| Norovirus | Envelope Structure GI.1 (Norwalk strain) | VLP | No | 33.3 | A | [9] |
| *Cryptosporidium parvum* | 17-kDa Antigen | Recomb. | Yes | 8.0 | B | [2] |
| *Cryptosporidium parvum* | 27-kDa Antigen | Recomb. | Yes | 14.5 | B | [2] |
| *Taenia solium* | T24H Antigen | Recomb. | Yes | 140.8 | B | [10] |
| *Schistosoma mansoni* | Sm25 Antigen | Recomb. | Yes | 14.4 | A | [11] |
| *Campylobacter jejuni* | P18 Antigen | Recomb. | Yes | 30.0 | B | [12] |
| *Campylobacter jejuni* | P39 Antigen | Recomb. | Yes | 28.8 | B | [12] |
| *Salmonella enteritidis* | Sero Enteriditis Group D | Native | No | 140.0 | B | [13] |
| *Salmonella typhimurium* | Sero typhimurium Group B | Native | No | 70.0 | B | [14] |
| *Chlamydia trachomatis* | CT-694 Antigen | Recomb. | Yes | 34.8 | A | [15] |
| *Chlamydia trachomatis* | Pgp3 Antigen | Recomb. | Yes | 139.0 | A | [15] |
| *Format: Recomb., recombinant; VLP, virus-like particle.  ^£^Coupling buffer: A, PBS, pH 7.2; B, 50 mM 2-(N-morpholino)ethanesulfonic acid, 0.85% NaCl, pH  5.0.  ^¶^Coupling amount: amount in µg per 12.5 million beads | | | | | | |

**References**

1. Poirier MJ, Moss DM, Feeser KR, Streit TG, Chang GJ, Whitney M, et al. Measuring Haitian children's exposure to chikungunya, dengue and malaria. Bulletin of the World Health Organization. 2016;94(11):817-25A. doi: 10.2471/BLT.16.173252. PubMed PMID: 27821884; PubMed Central PMCID: PMC5096354.

2. Moss DM, Priest JW, Hamlin K, Derado G, Herbein J, Petri WA, Jr., et al. Longitudinal evaluation of enteric protozoa in Haitian children by stool exam and multiplex serologic assay. The American journal of tropical medicine and hygiene. 2014;90(4):653-60. doi: 10.4269/ajtmh.13-0545. PubMed PMID: 24591430; PubMed Central PMCID: PMC3973509.

3. Rogier E, Wiegand R, Moss D, Priest J, Angov E, Dutta S, et al. Multiple comparisons analysis of serological data from an area of low Plasmodium falciparum transmission. Malar J. 2015;14:436. doi: 10.1186/s12936-015-0955-1. PubMed PMID: 26537125; PubMed Central PMCID: PMCPMC4634594.

4. Rogier E, Moss DM, Chard AN, Trinies V, Doumbia S, Freeman MC, et al. Evaluation of Immunoglobulin G Responses to Plasmodium falciparum and Plasmodium vivax in Malian School Children Using Multiplex Bead Assay. The American journal of tropical medicine and hygiene. 2016. doi: 10.4269/ajtmh.16-0476. PubMed PMID: 27895279.

5. Moss DM, Chard AN, Trinies V, Doumbia S, Freeman MC, Lammie PJ. Serological Responses to Filarial Antigens in Malian Children Attending Elementary Schools. The American journal of tropical medicine and hygiene. 2016. doi: 10.4269/ajtmh.16-0560. PubMed PMID: 27799641.

6. Rezaee MA, Rezaee A, Moazzeni SM, Salmanian AH, Yasuda Y, Tochikubo K, et al. Expression of Escherichia coli heat-labile enterotoxin B subunit (LTB) in Saccharomyces cerevisiae. J Microbiol. 2005;43(4):354-60. PubMed PMID: 16145550.

7. Lencer WI, Tsai B. The intracellular voyage of cholera toxin: going retro. Trends Biochem Sci. 2003;28(12):639-45. doi: 10.1016/j.tibs.2003.10.002. PubMed PMID: 14659695.

8. Chang GJ, Kuno G, Purdy DE, Davis BS. Recent advancement in flavivirus vaccine development. Expert Rev Vaccines. 2004;3(2):199-220. doi: 10.1586/14760584.3.2.199. PubMed PMID: 15056045.

9. Zambrano LD, Priest JW, Ivan E, Rusine J, Nagel C, Kirby M, et al. Use of Serologic Responses against Enteropathogens to Assess the Impact of a Point-of-Use Water Filter: A Randomized Controlled Trial in Western Province, Rwanda. The American journal of tropical medicine and hygiene. 2017. doi: 10.4269/ajtmh.16-1006. PubMed PMID: 28749764.

10. Handali S, Klarman M, Gaspard AN, Dong XF, Laborde R, Noh J, et al. Development and evaluation of a magnetic immunochromatographic test to detect Taenia solium, which causes taeniasis and neurocysticercosis in humans. Clin Vaccine Immunol. 2010;17(4):631-7. doi: 10.1128/CVI.00511-09. PubMed PMID: 20181766; PubMed Central PMCID: PMC2849338.

11. Won KY, Kanyi HM, Mwende FM, Wiegand RE, Goodhew EB, Priest JW, et al. Multiplex Serologic Assessment of Schistosomiasis in Western Kenya: Antibody Responses in Preschool Aged Children as a Measure of Reduced Transmission. The American journal of tropical medicine and hygiene. 2017;96(6):1460-7. doi: 10.4269/ajtmh.16-0665. PubMed PMID: 28719280; PubMed Central PMCID: PMCPMC5462587.

12. Schmidt-Ott R, Brass F, Scholz C, Werner C, Gross U. Improved serodiagnosis of Campylobacter jejuni infections using recombinant antigens. J Med Microbiol. 2005;54(Pt 8):761-7. doi: 10.1099/jmm.0.46040-0. PubMed PMID: 16014430.

13. Lopes G, Sousa C, Silva LR, Pinto E, Andrade PB, Bernardo J, et al. Can phlorotannins purified extracts constitute a novel pharmacological alternative for microbial infections with associated inflammatory conditions? PLoS One. 2012;7(2):e31145. doi: 10.1371/journal.pone.0031145. PubMed PMID: 22319609; PubMed Central PMCID: PMCPMC3271118.

14. Paolillo R, Romano Carratelli C, Sorrentino S, Mazzola N, Rizzo A. Immunomodulatory effects of Lactobacillus plantarum on human colon cancer cells. Int Immunopharmacol. 2009;9(11):1265-71. doi: 10.1016/j.intimp.2009.07.008. PubMed PMID: 19647100.

15. Goodhew EB, Priest JW, Moss DM, Zhong G, Munoz B, Mkocha H, et al. CT694 and pgp3 as serological tools for monitoring trachoma programs. PLoS neglected tropical diseases. 2012;6(11):e1873. doi: 10.1371/journal.pntd.0001873. PubMed PMID: 23133684; PubMed Central PMCID: PMCPMC3486877.
