# Supplementary material for: The impact of school water, sanitation, and hygiene improvements on infectious disease using serum antibody detection
Source: PLoS Negl Trop Dis. 2018 Apr 16;12(4):e0006418. doi: 10.1371/journal.pntd.0006418 (PMC5919668; doi:10.1371/journal.pntd.0006418)
Supplement: S2 Table — (DOCX) [file pntd.0006418.s002.docx]

| **S2 Table.** Prevalence of enteric and neglected tropical diseases among primary school children in Mali (n=800). | | |
| --- | --- | --- |
| **Pathogen** | **Antigen cutoff** | **Prevalence**  **n (%)** |
| Chikungunya | 509 | 43 (5.4%) |
| *Entamoeba histolytica* | 219 | 573 (71.7%) |
| *Giardia intestinalis* (VSP 5) | 444 | 420 (52.6%) |
| *Giardia intestinalis* (VSP 3) | 338 | 444 (55.6%) |
| *Plasmodium falciparum* (MSP 1_19_) | 222 | 626 (728.4%) |
| *Plasmodium falciparum* (MSP 1_42_) | 312 | 725 (90.7%) |
| *Plasmodium falciparum* (AMA 1) | 237 | 732 (91.6%) |
| *Plasmodium vivax* | 393 | 140 (17.5%) |
| *Brugia malayia* | 633 | 9 (1.1%) |
| *Wuchereria bancrofti* | 1350 | 2 (0.3%) |
| *Escherichia coli* | None | -- |
| *Vibrio cholera* | None | -- |
| Dengue 2 | 558 | 197 (24.7%) |
| Dengue 3 | 632 | 118 (14.8%) |
| Yellow fever | 1795 | 36 (4.5%) |
| Norovirus (St. Cloud) | None | -- |
| Norovirus (Sydney) | None | -- |
| Norovirus (Norwalk) | None | -- |
| *Cryptosporidium parvum* (17-kDa) | 960 | 385 (48.2%) |
| *Cryptosporidium parvum* (27-kDa) | 3591 | 345 (43.2%) |
| *Taenia solium* | 1068 | 65 (8.1%) |
| *Schistosoma mansoni* | 310 | 385 (48.2%) |
| *Campylobacter jejuni* (P18) | None | -- |
| *Campylobacter jejuni* (P39) | None | -- |
| *Salmonella enteritidis* | None | -- |
| *Salmonella typhimurium* | None | -- |
| *Chlamydia trachomatis* (CT-694) | None | -- |
| *Chlamydia trachomatis* (Pgp3) | None | -- |
