# Supplementary figures and images for: The impact of school water, sanitation, and hygiene improvements on infectious disease using serum antibody detection
Source: PLoS Negl Trop Dis. 2018 Apr 16;12(4):e0006418. doi: 10.1371/journal.pntd.0006418 (PMC5919668; doi:10.1371/journal.pntd.0006418)

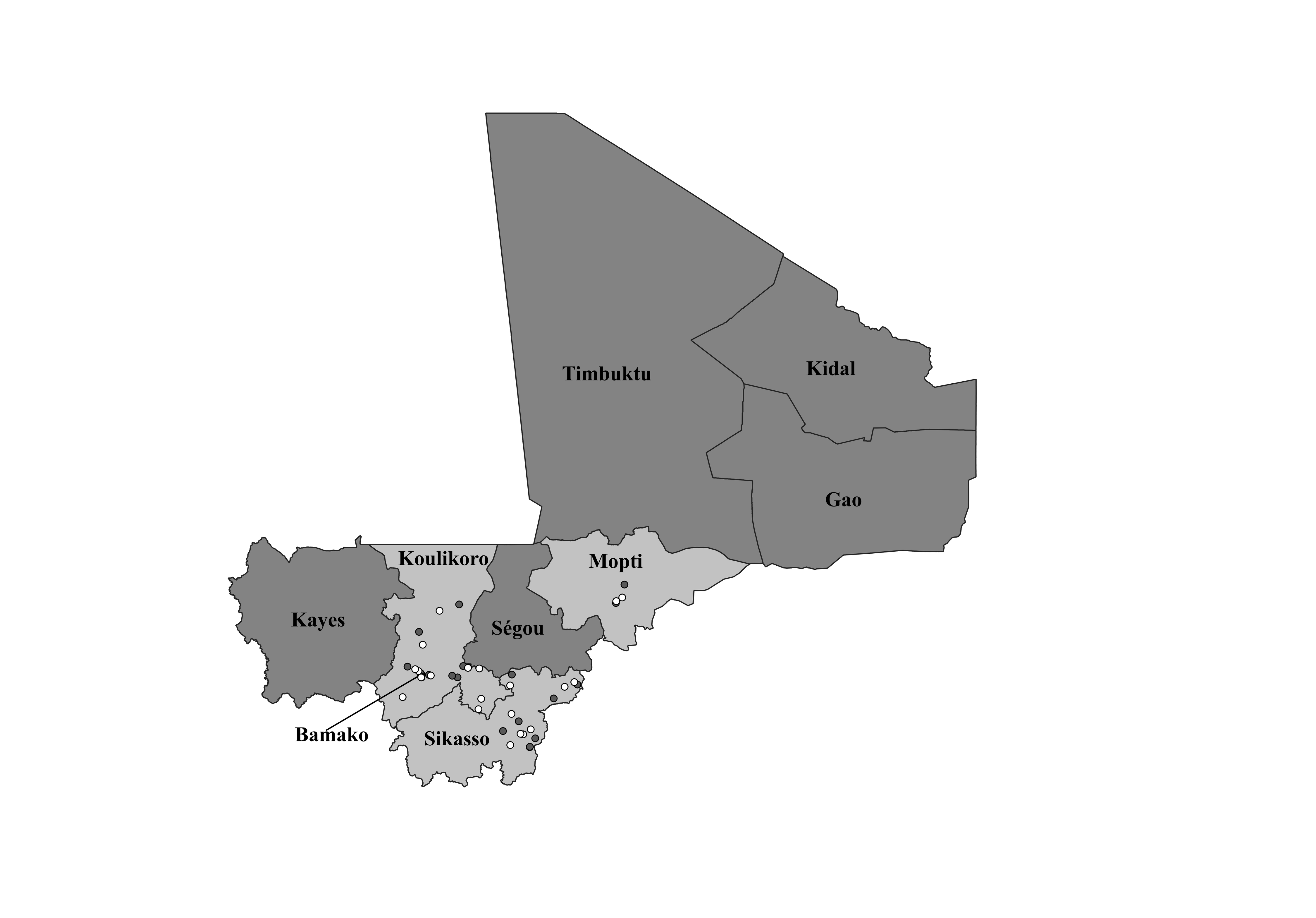

Supplement: S1 Fig — Light gray regions were included in study sample; dark gray regions were not included. Dark gray circles indicate location of beneficiary schools; white circles indicate location of comparison schools. (TIF) [file pntd.0006418.s003.tif]

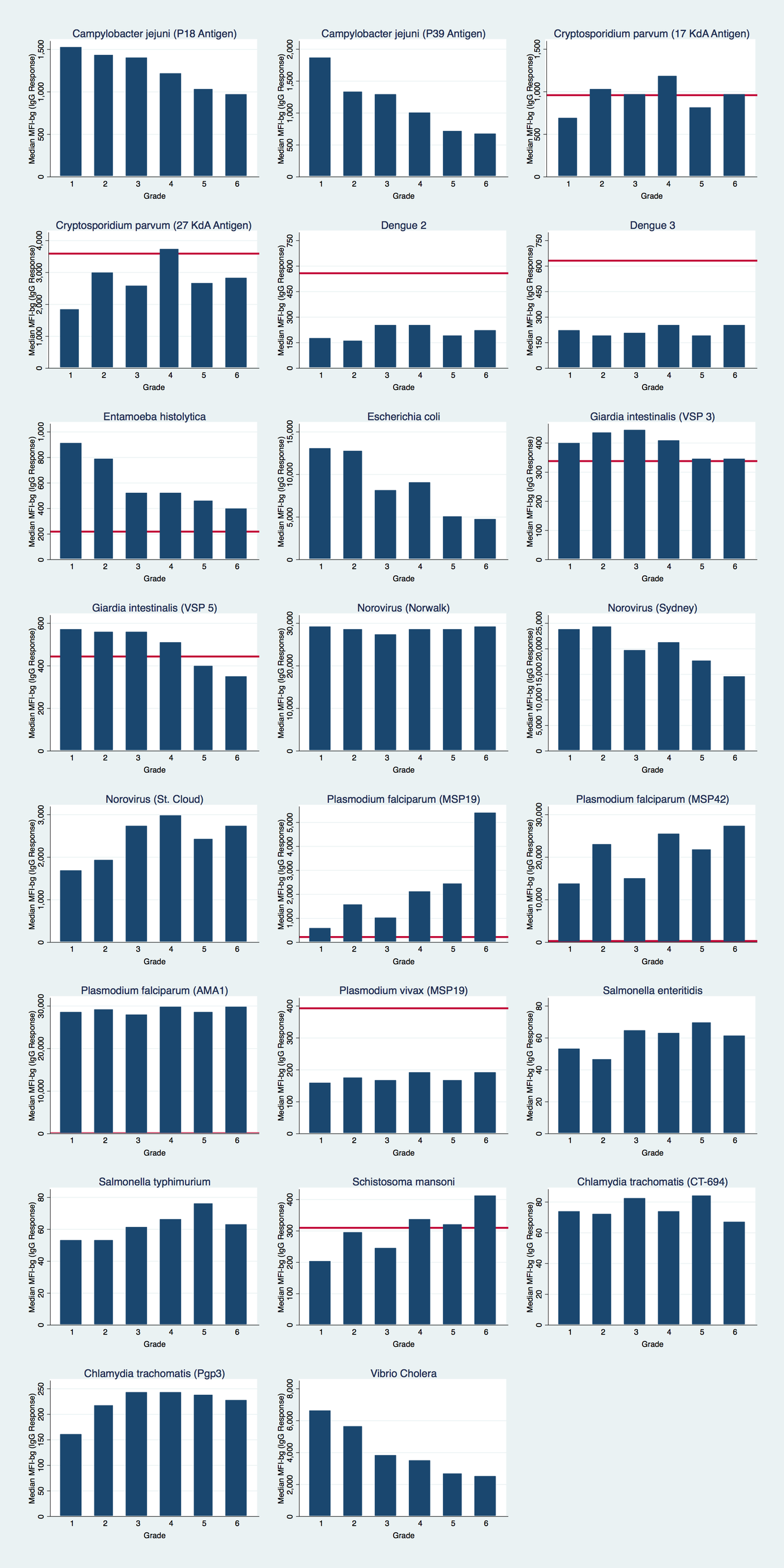

Supplement: S2 Fig — Red line indicates cut-off value for infection (if it exists). (TIF) [file pntd.0006418.s004.tif]
